# Editorial: Cognitive and Behavioral Features in ALS: Beyond Motor Impairment in ALS-FTD Spectrum Disorders

**DOI:** 10.3389/fneur.2022.891841

**Published:** 2022-06-14

**Authors:** Enrica Bersano, Umberto Manera, William Huynh

**Affiliations:** ^1^3rd Neurology Unit and Motor Neuron Disease Centre, Fondazione IRCCS Istituto Neurologico “Carlo Besta”, Milan, Italy; ^2^Department of Biomedical and Clinical Sciences “Luigi Sacco”, University of Milan, Milan, Italy; ^3^ALS Centre, Department of Neuroscience “Rita Levi Montalcini”, University of Torino, Turin, Italy; ^4^Brain and Mind Centre, University of Sydney, Camperdown, NSW, Australia; ^5^Prince of Wales Clinical School, University of New South Wales, Sydney, NSW, Australia

**Keywords:** cognitive and behavioral features, ALS (amyotrophic lateral sclerosis), frontotemporal dementia (FTD) spectrum, review and editing, biomarker, Motor neuron disease, MND

Over the last few decades, significant progress has been made in the understanding of cognitive and behavioral involvement in patients with amyotrophic lateral sclerosis (ALS). In this Frontiers Research Topic, the seven articles contributing to this special theme will help readers overcome the traditional concept of ALS being a disorder exclusively affecting only the motor system of the neuromuscular system, as well as frontotemporal dementia (FTD) being more than just a form of dementia, through novel neuroimaging, neuropsychological, and epidemiological studies.

Amongst the non-motor features of ALS, neuropsychiatric features have been less studied compared to cognitive dysfunction, especially in analyzing their frequency across the ALS-FTD spectrum of conditions. In their work on factors influencing non-motor impairment across the ALS-FTD spectrum, Devenney et al. identify a similar pattern of neuropsychiatric symptom severity in a mixed cohort of 250 ALS-FTD patients, demonstrating that diagnostic phenotype, disease duration, and global cognition scores were the strongest predictors of non-motor and neuropsychiatric impairments. Separately, disordered sleep and disrupted mood were also commonly found in pure motor phenotypes, thus suggesting a shared underlying pathophysiology involving the entire phenotypic spectrum.

Among the behavioral symptoms, apathy is the most frequently reported (detected in up to 70% of patients), although often concomitant and confused with depressed mood (1). Pain et al., based on a cohort of Italian patients, demonstrated the validity, reliability, and clinical utility of the ALS Depression Inventory-12 (ADI-12) questionnaire to assess depression, independent of motor disability and separately from cognitive features such as apathy. ALS-specific, motor-independent tools are required for the screening of depression to distinguish between treatable reactive/psychogenic forms and non-treatable conditions related to motor and cognitive dysfunction in patients with ALS-FTD spectrum disorders. Although therapeutic approaches to mood disorders are well-known, interventions for cognitive and behavioral symptoms as part of ALS care remain poorly defined and adopted. The first step to develop successful neuropsychological management is to focus on the presence of illness perceptions among patients and caregivers. To this effect, Caga et al., using the Brief Illness Perception Questionnaire (BIPQ), showed that a greater perceived cognitive and emotional impact on ALS increased the risk of behavioral changes and was also associated with more rapid disease progression, underscoring the importance of tailored emotional support as part of multidisciplinary ALS care.

Among symptoms described by patients as most socially embarrassing are episodes of involuntary laughter or crying (pseudobulbar affect). On this aspect, Tu et al. study confirmed a higher prevalence of pseudobulbar signs in bulbar onset ALS patients, applying the Center for Neurologic Study-Liability Scale (CNS-LS) as a validated tool. Using neuropsychological evaluation and magnetic resonance imaging (MRI), the authors also noted that pathological laughter correlated with executive dysfunction, brainstem volume reduction, and a lower fractional anisotropy of the superior cerebellar peduncles. Indeed, emotional lability could be underpinned by degeneration across distinct neural circuits with a critical role involving the brainstem.

Over the last several years, the acknowledgment of frontotemporal involvement in ALS has resulted in the extensive application of novel neuroimaging techniques (2). McKenna et al.'s review focused on insights gained from structural, metabolic, and functional neuroimaging studies that have improved the understanding of extra-motor disease burden in ALS phenotypes that were less frequently associated with dementia, i.e., primary lateral sclerosis, progressive muscular atrophy, or post poliomyelitis syndrome. The paper confirmed the heterogeneity of extra-motor pathology across the spectrum of motor neuron disease and highlights the role of neuroimaging in characterizing anatomical patterns observed in cognitive impairment and neurodegeneration *in vivo*.

Among neuroimaging tools, brain MRI has been commonly employed to monitor ALS disease progression (3). Another MRI technique, the Quantitative muscle MRI, represents a non-invasive tool that enables determination of the trajectory of pathogenic features at the muscle level in ALS patients, as demonstrated in a longitudinal study conducted by Paoletti et al. Their preliminary data showed that muscle damage in ALS is characterized by different MRI features in different phases of disease.

Although MRI can also be used in longitudinal studies, its utility to predict progression of disease has not been well-characterized, and is partly attributable to the lack of valid biomarkers in motor neuron disease. In this regard, Reyes-Leiva et al. reviewed the literature determining the role of different biomarkers of extra-motor neurodegeneration in ALS and their capability in identifying key pathophysiological changes at diagnosis (diagnostic biomarkers), to predict the risk or speed of progression (prognostic biomarkers), and to monitor response to therapy (therapeutic biomarkers) to characterize relevant aspects of disease pathophysiology (i.e., inflammation). Considering the possible difference in pathological mechanism underlying cognitive and behavioral features in ALS-FTD spectrum conditions, the availability of various bodily tissue/fluid, neuroimaging, and neurophysiological and genetic biomarkers would improve the recognition *in vivo* of different degrees of neurodegenerative and neuroinflammatory changes thereby allowing the development of targeted molecular treatment (3).

In conclusion, the present Frontier Research Topic delves into the extramotor features in motor neuron disease, expanding the traditional perspective of a pure neuromuscular disorder to a more complex motor and cognitive spectrum. The study of pathophysiological mechanisms and phenotype characterization in ALS-FTD will ultimately improve diagnosis, staging and management, thereby facilitating the design of personalized symptomatic treatment that include psychological support, as well as more comprehensive design of clinical trials.

## Author Contributions

All authors listed have made a substantial, direct, and intellectual contribution to the work and approved it for publication.

## Funding

WH was supported by the Lenity Foundation Fellowship, University of Sydney.

## Conflict of Interest

The authors declare that the research was conducted in the absence of any commercial or financial relationships that could be construed as a potential conflict of interest.

## Publisher's Note

All claims expressed in this article are solely those of the authors and do not necessarily represent those of their affiliated organizations, or those of the publisher, the editors and the reviewers. Any product that may be evaluated in this article, or claim that may be made by its manufacturer, is not guaranteed or endorsed by the publisher.
